# Successful Laparoscopic Management of Retroperitoneal Necrotizing Fasciitis: A Case Report

**DOI:** 10.7759/cureus.18677

**Published:** 2021-10-11

**Authors:** Monerh Bin Mosa, Ali Alyami, Saad Abumelha, Ali Alzahrani

**Affiliations:** 1 Division of Urology, Department of Surgery, Ministry of National Guard - Health Affairs, Riyadh, SAU; 2 College of Medicine, King Abdullah International Medical Research Center, Riyadh, SAU; 3 College of Medicine, King Saud Bin Abdulaziz University for Health Sciences, Riyadh, SAU; 4 Division of General Surgery, Department of Surgery, Ministry of National Guard - Health Affairs, Riyadh, SAU

**Keywords:** necrotizing fasciitis, retroperitoneum, laparoscopy, debridement, fournier's gangrene

## Abstract

Necrotizing fasciitis is a soft tissue infection characterized by rapid progression caused by gas-forming organisms. Retroperitoneal necrotizing fasciitis is a rare and fatal condition posing a diagnostic and management challenge. We present a case of a 28-year-old man who presented to the emergency department with a two-day history of diarrhea, right lower quadrant abdominal pain, dysuria, and a history of tender perianal swelling. A CT scan of the abdomen and pelvis showed extensive emphysema involving abdominopelvic retroperitoneal spaces. The patient underwent incision and drainage of the perianal area initially followed by diagnostic laparoscopy and drainage of retroperitoneal space. Early detection and extensive debridement of devitalized tissue are the cornerstones of the successful management of necrotizing fasciitis. This is the first case to report laparoscopic management of retroperitoneal necrotizing fasciitis.

## Introduction

Necrotizing fasciitis (NF) is a life-threatening, rapidly progressing soft-tissue infection. NF typically occurs in immunocompromised patients with few cases reported in healthy individuals. It can affect any body part but is commonly seen in the extremities, perineum, genitalia, and to a lesser extent, the chest and abdomen [1]. NF has an estimated incidence of 1,000 cases per year with high associated morbidity and mortality rates. Retroperitoneal necrotizing fasciitis (RNF) is a distinctive rare entity with few cases reported in the literature [2]. While NF is primarily a clinical diagnosis, identifying retroperitoneal extension is challenging as the presenting symptoms develop insidiously and are non-specific. The key elements in managing necrotizing infections are early identification, extensive debridement, and targeted antimicrobial therapy [1].

## Case presentation

We present a case of a 28-year-old immunocompetent male with a history of mild asthma, who was on bronchodilators as needed. He presented to the emergency department complaining of a two-day history of perianal pain and swelling followed by right lower quadrant abdominal pain, diarrhea, and dysuria. The patient denied a history of fever or other systematic symptoms. During the examination, the patient looked well and alert, but he was anxious and tachycardic with a heart rate reaching 128 beats per minute. Other vital signs were normal. Abdominal examination revealed a mildly distended abdomen with severe right lower quadrant tenderness, but there was no guarding. On perianal examination, a small induration was noted on the left side of the gluteal region extending to the perineum with overlying skin erythema. An extensive subcutaneous crepitation was felt on the perineum reaching the left scrotum and medial upper thigh. The digital rectal exam was unremarkable of any masses, collections, cavitation, or bleeding.

**Figure 1:**
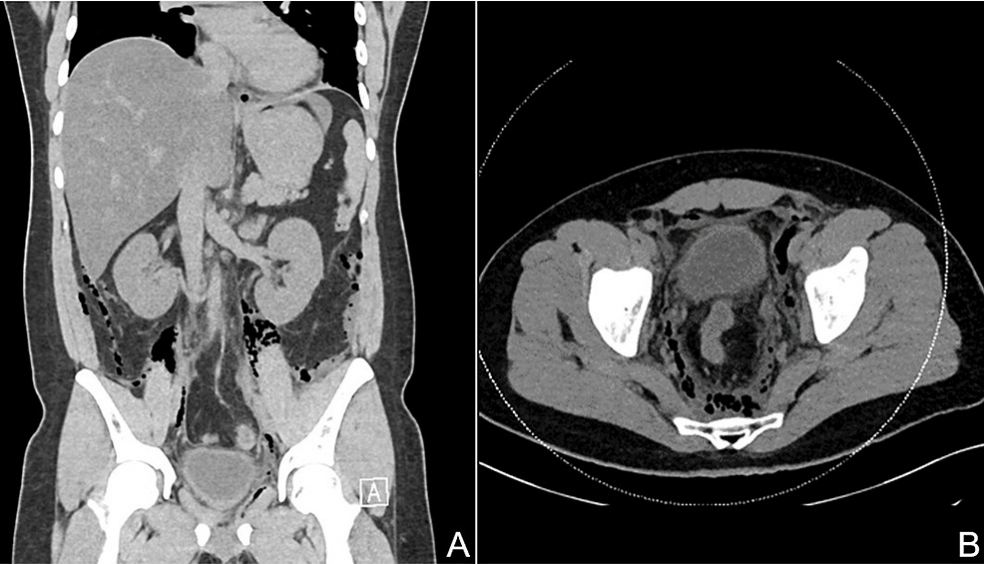
A non-enhanced CT scan of the abdomen and pelvis showing extensive emphysema of the extraperitoneal spaces. (A) A coronal image showing upward extension of retroperitoneal gas. (B) An axial section of the pelvis with widespread emphysema.

Blood investigations showed leukocytosis (white blood cell count: 14.20 x 1,000/mm^3^) with neutrophil predominance, acute kidney injury (serum creatinine: 172 umol/L), hyperglycemia (glucose: 10.5 mmol/L), deranged serum electrolytes (sodium: 130 mmol/L, potassium: 3.2 mmol/L, and chloride: 88 mmol/L), and metabolic acidosis with a high anion gap due to lactic acidosis. A computed tomography (CT) of the abdomen and pelvis was performed by the emergency physician before consulting the surgical team. The images showed edema, fat stranding, and fascial thickening of the perineum with extensive emphysema extending through the pelvic, prevesical, and posterior pararenal retroperitoneal spaces bilaterally, and into the scrotum and upper inner thighs distally (Figure 1).

Incidental finding of bilateral patchy consolidation on the lower cuts of the lungs was suggestive of asymptomatic coronavirus disease 2019 (COVID-19) pneumonia.

While the initial images did not identify a source of the infection, rectal pathology was suspected. The patient received broad-spectrum antibiotics, piperacillin/tazobactam, and vancomycin for a provisional diagnosis of Fournier’s gangrene due to perianal abscess. After the initial assessment, the patient started spiking fever (temperature 38.4°C). The decision was made to take the patient urgently for debridement of the perianal region. Bilateral vertical incisions were made to access the ischiorectal fossae. On the left side, the devitalized tissue was limited to a small area of the subcutaneous, and only minimal pus was drained and sent for cultures. The subcutaneous tissue on the right side was healthy. As the intraoperative findings were localized and debrided thoroughly, we packed the wounds with iodine-soaked iodoform and opted to terminate the surgery as the patient became tachypneic, hypotensive, and more acidotic. The patient was shifted to an intensive care unit (ICU) postoperatively. A pelvic CT with rectal contrast was obtained the next morning, to rule out rectal pathology, which showed no rectal or colonic contrast leak with stable inflammatory features observed in the previous imaging. Postoperatively, the antimicrobial therapies were upgraded to meropenem, metronidazole, linezolid, and caspofungin, and then adjusted according to cultures’ sensitivity, which grew *Streptococcus anginosus* and *Escherichia coli*. The fever subsided after 48 hours and the patient’s biochemical markers were improving.

**Figure 2:**
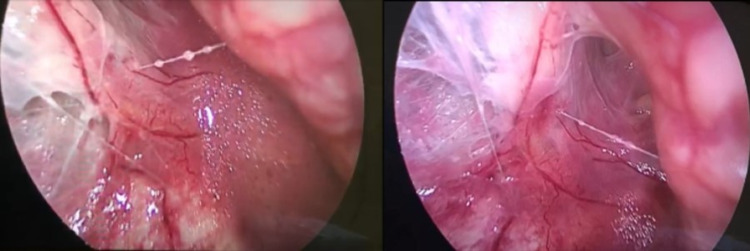
Laparoscopic intraoperative images.

On the fourth day of admission, the patient was taken for a second look at the perianal wounds and bilateral diagnostic laparoscopy of the retroperitoneum (Figure 2).

The patient was placed in the right lateral decubitus position with the bed flexed at the waistline. After securing 10 mm and 5 mm ports, blunt dissection was done with care so as not to violate the peritoneum until reaching the hypochondrium superiorly and the internal inguinal ring inferiorly. A moderate amount of pus was noted. Multiple irrigation cycles were done until the tissues looked healthy. A 15Fr Jackson-Pratt drain was inserted and the ports removed and the steps repeated on the left side. The patient's condition improved postoperatively, substantiating no further trips to the operating room. Wound dressing was performed regularly at the bedside. The drains were draining a small amount of fluid and were removed shortly. A CT scan of the abdomen and pelvis was repeated on the 13th day of admission, which showed the resolution of the signs of RNF. The patient was discharged home after 21 days of admission, and he was faring well during outpatient follow-up for wound management for a month after discharge.

## Discussion

Necrotizing soft tissue infections are estimated to progress at a rate of 2 cm per hour, resulting in significant local tissue destruction and rapid progression of sepsis to end-organ damage due to shock; therefore, early diagnosis is crucial to increase the chances of survival [3]. Although NF is a clinical diagnosis, retroperitoneal involvement is difficult to identify as it presents with signs and symptoms of acute abdomen. In our case, the patient had non-specific abdominal complaints with a history of perianal swelling directing the examination to the perianal and genital area revealing signs of NF; however, without the imaging, the diagnosis of RNF would have been delayed, leading to a less fortunate outcome. Obtaining an image at the initial presentation is valuable to assess the extent of the disease and guide the surgical intervention, yet surgical intervention should not be delayed in the process [4]. A CT scan is superior to other modalities given the limited information gained from ultrasonography and plain radiographs, and limited accessibility of magnetic resonance imaging on an urgent basis. CT of necrotizing infection usually demonstrates asymmetrical fascial thickening, diffuse collection, and emphysema dissecting through the fascial planes; even though the presence of gas in the retroperitoneum is highly suggestive of RNF, its absence does not preclude the diagnosis [5].

We reviewed all cases of RNF reported over the past three decades. All patients were managed with debridement of the primary infection source, commonly, perianal abscess proceeded with laparotomy. The retroperitoneal involvement in most cases was discovered intraoperatively. As our patient was young and healthy, we elected to proceed with diagnostic laparoscopy of the retroperitoneum. We simulated the approach of laparoscopic retroperitoneal nephrectomy to avoid the risk of introducing the infection into the peritoneum, and since the patient was caught in the early stages of the infection as evident during the perianal debridement, our goal was to limit the morbidity associated with a major surgical intervention.

NF has high mortality rates reaching up to 50% of the cases, and retroperitoneal involvement was considered a feature of the advanced fatal disease [1,6]. Currently, many cases are being reported with successful outcomes even in patients with multiple co-morbidities and suppressed immunity; however, survival is frequently associated with severe morbidity due to the disease process and complications of the treatment. Such as seen in a report of a healthy middle-aged male who developed RNF with no identifiable risk factors. The patient was initially managed successfully with laparotomy and discharged home to present later with bleeding at the surgical wound site due to arterial bleed requiring readmission, blood transfusion, and angioembolization [7]. In another report, a young male had RNF following blunt trauma. Due to the patient’s immunocompromised status, he had a severe disease difficult to control, requiring multiple aggressive surgical debridements resulting in lower limb amputation and subsequent reconstruction [8]. In our report, the patient has fortunately survived with no major consequences. This can be attributed to his young age, immune status, and early recognition and delivery of treatment.

## Conclusions

RNF is a rare and fatal condition. Early diagnosis, intensive debridement, and appropriate antimicrobial therapy are the fundamentals of management. Effective surgical treatment can be achieved laparoscopically in the hands of an experienced surgeon. Laparoscopy can be a feasible option in young healthy individuals and patients who present at an early stage of the disease. The benefits of a minimally invasive approach should be weighed against the risk of suboptimal control of the infectious source, particularly, in cases of inexperience.
